# Development of a digital tool to support overview in complex patient cases: Which information elements support the clinical workflow?

**DOI:** 10.1186/s12913-026-14466-6

**Published:** 2026-04-11

**Authors:** Amanda Emén, Marte Broks, Torbjørn Torsvik, Gro Rosvold Berntsen, Aslak Steinsbekk

**Affiliations:** 1 Department of Public Health and Nursing, Faculty of Medicine and Health Sciences, Norwegian University of Science and Technology (NTNU), Trondheim, 7491 Norway; 2 Norwegian Centre for E-health Research, Tromsø, Norway; 3 Institute of Community Medicine, UiT The Arctic University of Norway, Tromsø, Norway

**Keywords:** Complex care needs, Person-centered, Patient overview, Usability, Workflow

## Abstract

**Aim:**

This study aimed to describe the development and usability testing of the digital tool DigiTeam to support physicians’ clinical workflow and identify key information elements to support clinicians in managing complex patient cases.

**Method:**

This qualitative study was guided by user-centered principles with observations and interviews of physicians who tested different versions of DigiTeam. Three patients volunteered to have their full electronic health records (EHR) from the following sources: general practice, hospital, and, if relevant, municipal health and social care services, included in DigiTeam. The prototype was developed to gain knowledge on how to present complex patient case information in EHR. The key clinical principles guiding the development were to support person-centered, integrated, and proactive care. The design principles included providing overviews before details, summarizing information, and using timelines. The data were continuously analyzed to support iterative development.

**Results:**

Seventeen physicians participated, and five versions were developed. The start page was simplified and at the end included only three key information elements that supported a quick overview: patient voice, medical history summary, and main health problems with healthcare contacts displayed on a timeline. The participants highly valued the prominent inclusion of the patient’s voice, which was maintained throughout. The elements on the start page were clickable, directed to pages with summarized information, and hyperlinks to clinical notes for further details and verification of the summarized information. The participants found the summarized information valuable but initially too text-heavy, resulting in versions with less text while retaining essential details.

**Conclusion:**

It was possible to develop a digital tool that supported person-centered, integrated, and proactive care by summarizing information to provide an overview of three main information elements: what matters to the patient, the main health problems, and medical history, with easy ways to obtain more detailed information. The next step is to test the effect of DigiTeam in a randomized controlled trial.

**Clinical trial number:**

Not applicable.

**Supplementary Information:**

The online version contains supplementary material available at 10.1186/s12913-026-14466-6.

## Background

Person-centered, integrated, and proactive care are essential goals in high-quality healthcare for individuals with complex care needs [1]. Achieving these goals requires that health professionals have access to all relevant information to address the full spectrum of a patient’s needs and situation. This information includes the patient’s context and what matters to the patient, ongoing activities and medical problems, and who is involved in their care [1]. By viewing care as a longitudinal process, where the different tasks are seen as one in a series of events instead of a task-by-task approach for one disease, it can be better integrated and coordinated [1]. Access to this comprehensive information is also crucial for proactive care, which involves carrying out holistic assessments, developing personalized care and support plans, delivering coordinated multi-professional interventions to address a person’s range of needs, and providing a clear plan for continuity of care [2, 3]. This approach is particularly crucial when multiple health problems and complex, long-term care needs are involved, requiring the collaboration of numerous health professionals and organizations [4].

Clinicians often spend a significant amount of time obtaining a comprehensive overview of complex patient case information, including the patient’s wishes, context, and medical history. This involves searching and summarizing information from multiple sources to identify patterns, current health problems, and treatment plans [5]. When numerous notes are spread across various electronic or paper-based systems, retrieving this information can be challenging due to a lack of access to all the information, information overload, ineffective presentation, and fragmented displays requiring additional clicks [6–8]. Insufficient support for clinicians’ information needs can lead to high cognitive load, errors, and missing information [6]. Therefore, tools that help clinicians quickly access essential information and align with their workflow are necessary, especially in time-constrained environments [5, 9, 10].

Improving interoperability has been found to have some effect on quality of care, like patient safety events and medication safety [11]. Furthermore, solutions that collect and summarize large amounts of clinically relevant information have been reported previously [12] and can support clinical workflows [12, 13]. Several studies have reported features in digital solutions that support quick access and overviews of patient information, such as visual presentation of information over time via timelines and graphs [14–21], summarizing or reorganizing information displayed on one page [15–17, 19–22], and information organized according to health problems [15–17, 21–25]. User feedback indicates that such features can help understand the patient’s history [16, 21], retrieve clinical information more easily [15] and faster [18, 23], and recognize trends [20]. Such features can also have the potential to reduce cognitive load and information overload [22], make it easier to identify active problems that include nonmedical problems [24], and support clinicians’ workflow [16, 17, 21]. Although clinicians prefer to access information overviews, complex long-term care histories are challenging, as these histories are often unique to each patient and lack standardization [26].

Ensuring the availability of essential information is another problem, since many electronic health records lack interoperability and are stored in silos that are challenging for those outside the organization to access [1]. This has been described as fragmenting the patient information in a hospital, leading to a lack of mutual knowledge of the patient’s situation among those involved in care [5]. Some studies have investigated how to retrieve information from different sources [27–29]. As more information leads to more work in gaining an overview of the information, they have also worked on how to present this information, e.g., by presenting all the information in the same view [21]. Testers have given positive feedback, as it can provide a comprehensive view of all available information [28], are useful when meeting a new patient [21], provide a better understanding of medical history [21, 27], and reduce workload and missed information [29].

Involving patients in providing their perspective on their health situation, values, and needs is important for a broader understanding of patient’s situation in person-centered care [30]. This includes information about what matters to the patient [1], what support the patient receives from informal caregivers, their social and family environment, personal goals and preferences, and past nonmedical experiences that impact their health [31]. Clinicians highlight the importance of such information when tailoring care to patients’ needs [32, 33]. Nevertheless, most systems lack a designated area for recording and presenting such contextual information about the patient [32, 34]. The proposed digital solutions to address this have included features that present the patient’s values, goals, and preferences [35–37], patient-reported outcomes on symptoms and disease-specific measures and goals [18, 38–44], and care planning to support proactive care [45]. These features have received positive responses from both clinicians and patients, as they can support talking about what matters most [38], tracking patients’ symptoms and goals [44], making clinical decisions and discussions [39], and communicating about preferences and needs [41].

In summary, existing solutions have mainly focused on one or a few of the areas described above, such as providing clinicians with an overview of medical information in specific settings or for specific diseases [18–20, 27], dashboards presenting the patient’s perspective related to a specific disease [38–40, 43, 44], presenting a solution for the patient’s values, goals, and preferences [35–37], and collecting notes and presenting summaries from several sources [21]. A few studies have focused on providing a clinical overview of complex patient information [15–17], for children with complex care needs [24], and identifying risks and care planning [45]. No study has specifically identified the key information elements necessary to help clinicians gain an overview of concurrent problems and the longitudinal care process in complex patient cases for decision-making that aligns with patient-centered, integrated, and proactive care principles.

Therefore, this study aimed to describe the development and usability testing of the digital tool ‘DigiTeam’ to support physicians’ clinical workflow and identify key information elements to support clinicians in managing complex patient cases.

## Method

### Study design

This study used qualitative methods, including observation and interviews, guided by user-centered principles [46]. Data collection took place between June 2023 and May 2024.

### Ethics and privacy concerns

This study was part of a larger project, and approval was waived by the Regional Committee for Medical and Health Research Ethics (application #: 251632) and approved by the data protection officer at the University Hospital of North Norway (# 2760). The participants and patients received written consent and voluntary participation and information about the study before the data collection.

### Description of DigiTeam

The background for the development of DigiTeam was based on research on how to improve the quality of care for patients with long-term and complex healthcare needs. This was summarised in a scoping and systematic intervention review [1], finding that clinicians lack good solutions to support them in providing person-centered, integrated, and proactive care. To address this gap, this work sought to identify key information elements that support physicians in delivering such care. This was done by developing a prototype, DigiTeam, to identify and conduct iterative testing of these elements, and to be able to test the effect of such a solution on the quality of care. The version of DigiTeam presented below will be used as the intervention in an experimental randomized controlled trial. The intention was not to develop a product for immediate implementation in care systems. Instead, the knowledge derived from this work will be communicated to vendors and buyers of electronic health record systems to improve their products.

To guide the development, comprehensive discussions were held within the research group, informed by previous research [1, 47], to identify the key quality of care principles that would support person-centred, integrated, and proactive care. The ones chosen were: (1) Giving access to all relevant information by providing it from several sources, (2) Ensure that the care is tailored to the patient by highlight the patient’s social and contextual situation, and what is important for the patient (“what matters to you”), (3) Increase awareness of involved actors (health professionals, next of kin and others involved) by presenting who have been involved in the care of the main health problems, and (4) Help focusing the efforts and coordinate the care of the actors involved by providing an overview of the main health problems and all ongoing care plans.

The key design principle followed was “Overview first, zoom and filter, then details-on-demand” [48]. This visual information-seeking mantra, introduced by Shneiderman, advocates a user-centered approach to interface design that supports intuitive exploration of complex data collections. Initially, users are presented with a comprehensive overview to establish context and orientation. They can then zoom in on areas of interest and apply filters to exclude irrelevant data, enabling focused analysis. Finally, detailed information is made accessible on demand.

Throughout the process, the development focused on translating the guiding key quality of care and design principles into usable features by changing the layout, content, and functional solutions. This study is part of a larger project that also aims to gain knowledge on the information needs of health professionals in comprehensive care teams for a digital tool to support person-centered, integrated, and proactive care, which has played a crucial role in the development of DigiTeam.

The description of DigiTeam presented below is the final version.

**Fig. 1:**
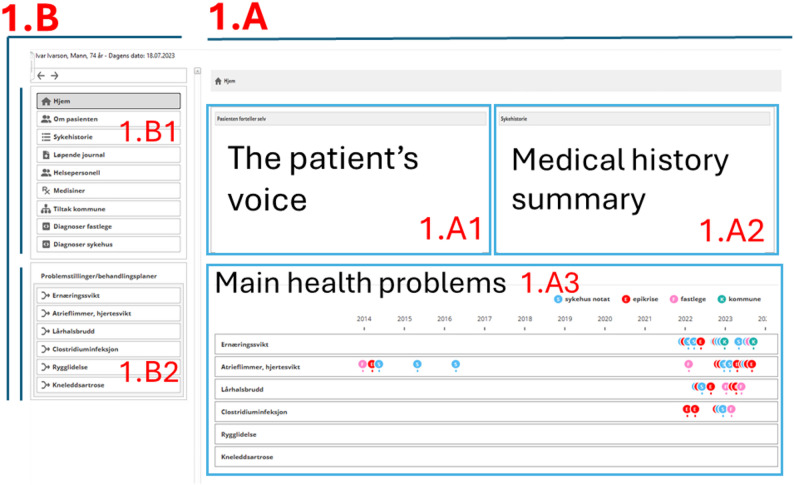
The start page of DigiTeam. Section  **1.A** shows the three main elements used to provide an overview: the patient’s voice (**1.A1**), a medical history summary (**1.A2**), and the health problem timeline (**1.A3**). Section **1.B** shows the sidebar menu

**The main part of the start page** (Fig. 1.A) includes three main information elements (section B). All the parts are clickable to obtain more detailed information, as described below. The three elements contain brief information to provide an overview of the following:

- **The patient’s voice** (Fig. 1. A1): A summary of the answers to the following questions: “What is important for you right now? “, “What matters to you in your life?” and “What do you want your care team to know about your history?” based on previous research [49].
- **A medical history summary**: Curated summary of the patient’s medical history (1.A2).
- **A health problem list with a timeline**: Main health problems with the main healthcare contacts for each problem on a timeline. The different colors show notes written in general practice (GP), municipal health and social care services, and at the hospital (1.A3) that were included in the health problem summary (described below).

**Fig. 2:**
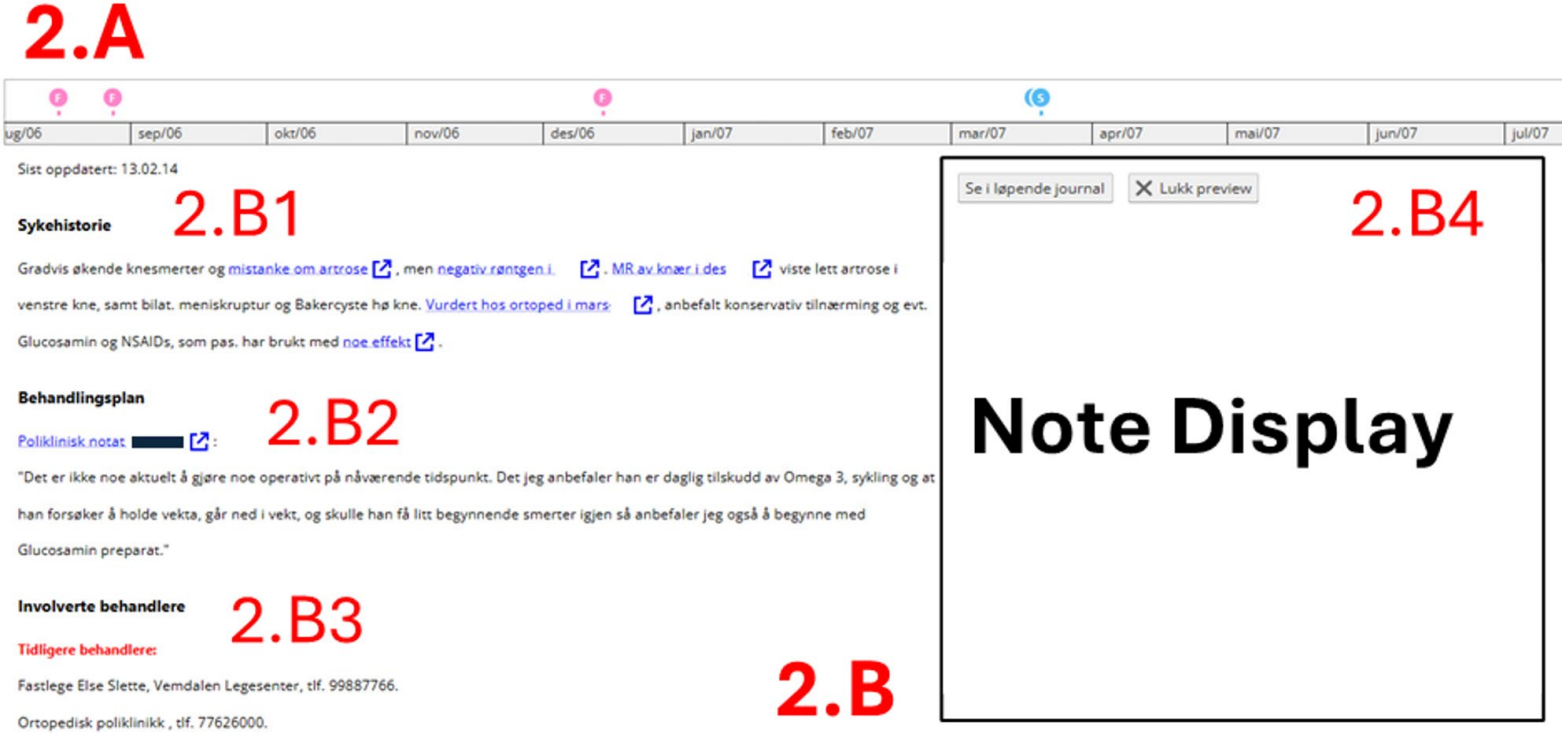
Example of a summary page. The summary pages include blue hyperlinks to access details, and when clicking on the link, the note is displayed on the right side (**2.B4**). This specific page is a health problem summary page for a specific health problem: Timeline with health care contacts on the top (**2.A**), and the summary (**2.B**) consisting of disease history (**2.B1**), treatment plan (**2.B2**), and involved health professionals (**2.B3**)

**The sidebar menu** (Fig. 1, section 1.B) is divided into two main areas (1.B1 and 1.B2). On the top (1.B1), there are choices for (in descending order):

1. **About the patient**: A summary page including critical information, the patient’s voice, special needs, family/social, next of kin, hereditary diseases, allergies, municipal health and social care services, housing/living situation, occupational history, and smoking/alcohol/stimulants. Hyperlinks refer to medical notes. This page can also be accessed by clicking on the patient voice box on the start page (1.A1).
2. **List of notes**: A list of all available notes from the GP, hospital, and municipal health and social care services merged on the same timeline. The note types and dates are listed in order on the left side, starting from the most recent one, and the corresponding full texts of the notes are listed in the same order on the right side (Additional File 1, Figure S3).
3. **Medical history**: A chronological descending list (latest on top) of the main events related to diseases and functional status (Additional File 1, Figure S4). Hyperlinks refer to a medical note or a health problem summary (described below).
4. **Involved professionals**: A list of current and previous professionals or departments involved in the care organized on health problems. This page does not include hyperlinks to notes. While the idea of adding links to each provider’s notes was considered, it was ultimately deemed to offer limited value.
5. **Medication list**: This list is based on information from the GP and hospital, organized according to current medication, medication to be used as needed (PRN), and past medication, with hyperlinks to the notes relevant for start, change, and stop (Additional File 1, Figure S5).
6. **Municipal health and social care services**: A separate page for municipal health and social home and institutional care services (e.g. nursing homes, residential care, home healthcare services). This page presents action plans for different care areas visualized on timelines, showing when a service was created, changed, or discontinued (Additional File 1, Figure S6).
7. **Diagnoses from GPs and hospitals**: Listed and organized chronologically, starting with the most recent diagnosis.
8. **Health problem summary pages** (Fig. 2): Accessed via the list on the start page (Fig. 1. A1) and in the sidebar menu (Fig. 1.B2). The upper part provides a timeline (2.A) marking when the most relevant notes were written for the identified health problem. The pages include a curated summary of the disease history (2.B1), treatment plan (2.B2), and involved health professionals (2.B3) based on notes from all sources. The blue hyperlinks provide the original note where the information originated and can be opened for more details and the possibility of verifying the summarized information. Clicking on a hyperlink opens the note on the right side (2.B4).

The content on the summary pages and other features was mainly extracted and written manually by one of the authors (MB), who has worked as a GP and is now a physician in a comprehensive care team set up by a hospital and municipalities.

### Participants

The inclusion criterion for participants in this study was physicians with experience in clinical work, aiming for variation in the type of professional experience, specialization, and age. The recruitment started by using the authors’ networks, and eligible physicians were contacted to inform them about the study and ask about their interest in participating. After that, the recruited physicians were asked if they knew any other potential participants. All recruited participants received a consent form via mail and were offered oral clarification before the test session.

### Test setting and procedure

In the initial 9 test sessions (6 of these were used as data in a medical student thesis with the first (AE) and last (AS) authors as supervisors [50]), the task was to try out the program with no structured information on how to use DigiTeam. For all subsequent tests, a structured introduction was given before the task. The task was to use DigiTeam with patient information from one of the patient cases (more details below) to (1) summarize the patient’s past medical history and current health status, (2) write a note about what is important for the patient, and (3) outline plans for further actions.

Each participant performed one user test at one point in time, with one test leader attending either alone or with another observing the test digitally via Microsoft Teams. The test setting was a separate room with a desk and two screens, where the test leader sat to the side and slightly behind the participant. Screen one displayed DigiTeam, and screen two had a Microsoft Word document with the task and a place to write the answers. The test leader’s role was to give instructions to the participants, observe the use of the program, and lead the interviews.

Three patients consented to have their histories as the basis for the three patient cases that were used for the tasks. These patient cases were based on notes from the hospital, the GP, and municipal health and social care services. These notes were deidentified by changing the names of the patient, the location, healthcare providers, hospitals, and GP clinic. The context of the patient cases was in general practice for two of the cases and a hospital for one of the cases. All the patients had complex chronic conditions with social and contextual challenges that influenced their health situation. The patient cases also had a large number of notes, in PDF format, ranging from approximately 300 to 3050 pages, and covered periods from 1 to 27 years, sourced from GPs, hospitals, and municipal health and social care services. The patients were also interviewed to gain insights into their care experiences and what matters to them.

### Data collection

Data collection involved observations of the user tests, with participants being encouraged to think aloud to verbalize immediate reactions and thoughts, and semi-structured interviews following the test. The screen activities and audio of both the test sessions and the interviews were recorded.

The observations focused on how the participants navigated DigiTeam, how they used the available features, whether any technical problems were encountered, and the search for information. Observation notes were written during the testing by the test leaders to be used as prompts in the interviews after the test. In addition, more detailed notes were written after the test session while looking at the screen recordings to ensure correct representation.

The semi-structured interviews conducted after the tests had open-ended questions about the overall impressions of DigiTeam, what features the participants used to solve the case, the usefulness of the different features, suggestions for improvements regarding content and layout, and features they usually use in clinical practice that were not included in DigiTeam. The observation notes were used as prompts, emphasizing moments when the participants seemed to struggle. The participants were also asked to compare with the systems they use in daily practice and provide examples. The participants were encouraged to review and use DigiTeam during the interview to illustrate their points. The details of the interview guide (Additional file 2) were slightly modified throughout the data collection process to include previous findings and adapt to the different versions of DigiTeam.

### Analysis

The development of DigiTeam was an iterative process informed by the participants and discussions in the research group. After each test session, the test leaders informed the program developer (TT) about the findings, and the research group discussed further ideas.

The analysis was an ongoing iterative process between data collection from the tests, analysis, and development of DigiTeam. The analysis was conducted by the research group led by the authors who performed the tests and the program developer to determine the next steps for development based on the findings.

In the final phase, all the accumulated data, including data from the ongoing analysis during the development of DigiTeam, were consolidated and analyzed mainly by the first author, with regular discussions with the last author and intermittent meetings with all the authors. The first author transcribed the interviews and “think aloud” sessions verbatim and took detailed notes from the video-recorded observations to describe how the participants used DigiTeam. The software programs NVivo version 14 (Lumivero https://lumivero.com/, Denver, USA) and Microsoft Word were used to manage the data during the analysis. Several subversions of DigiTeam that had only minor changes were merged and considered one version, whereas when a major change was made, it was defined as a new version. Minor changes were small-scale with bug fixes and smaller changes in the features, and major changes involved substantial modifications such as changes to the layout and adding, changing, or removing features.

Each transcript was first analyzed independently by extracting relevant text segments and organizing them into temporary themes (summaries, patient voice, program usage, and others). The extracts for each transcript were then grouped and summarized based on the version of DigiTeam so that each version had a summary for each theme. The final analysis considered these summaries across different versions and the changes made in DigiTeam. Based on the study aim and the main features of DigiTeam, the major themes were identified and agreed upon in the research group. Representative extracts from the transcripts were chosen and translated into English, and the extracts are marked with the participant’s gender and main type of professional experience. Finally, after thorough work with the results section, the first author reread each transcript to ensure that important details and perspectives were considered. Regular discussions in the research group were performed throughout the analysis and development process.

## Results

### Participant characteristics

Seventeen physicians (14 females), aged between 28 and 56 years, participated in the formal testing. The main types of professional experience were hospital departments (*N* = 8), comprehensive care teams (*N* = 2), emergency care (*N* = 2), and general practice (*N* = 5). The number of years in clinical practice ranged between 1 and 16. The test session and interview lasted, on average, 48 min (ranging from 20 to 104 min).

Five versions of DigiTeam were developed. The initial version was tested by 6 participants and then by 3, 5, and 3 participants for the following versions. The final version was the fifth version presented above, and this was not tested since most of the features in that version were either a combination of those already tested or refinements of the features.

The main findings were categorized into three themes that concerned central areas both in the purpose of DigiTeam and in what the participants mostly commented on: (1) *workflow*, which concerns how the tool was used, (2) *person-centered care*, which concerns the patient’s voice and context, and (3) *support integrated and proactive care*, which concerns gaining an overview of medical and health information over time from several sources (the GP, hospital and municipal health and social care services).

### Workflow

The premise from the beginning was to develop a solution that facilitates physicians’ workflow in retrieving patient information before the first meeting in a non-acute situation. This was also important for the participants, who frequently commented on the challenges of gaining an overview of a patient’s situation in clinical practice, both in general practice and hospital care. Overall, the comments were to simplify the layout, reduce the amount of text while keeping essential information, and minimize complicated features.

Still, most participants described the different versions as intuitive to use and easy to navigate when asked to summarize their experience after the test.

The observations revealed that most participants across all the versions started to read the patient’s voice and the information about the patient. In the third and fourth versions, the participants continued by viewing the health problem list and timeline to identify the patient’s main health problems. However, some participants did the opposite and started with the health problem timeline and subsequently read about the patient and the patient’s voice.

*The ‘Overview’ was very good for visual presentation of the patient’s diseases and getting a quick impression of what we need to consider here. I used it a lot. I used ‘About the patient’ a lot to lay out the actual plan. I used the plan overview [health problem summary] to read the latest status for the diagnoses that I considered important. I didn’t use ‘diagnoses’ or ‘medications’ much. And then I used the note list*,* both the shortcut to open the note list and to open discharge summaries to find information quickly. *(Male, Hospital)

The participants talked about finding notes with summarized information in their everyday practices. The search for these types of notes, such as admission and discharge notes, was performed by all the participants across the versions. Some mainly used the note list to find information, whereas others combined the note list with other features in DigiTeam.

*I mostly used the note list*,* partly because that’s what I’m used to. And by doing that*,* I know where the information comes from […]. I could have used it* [other features in the program] *a bit more if I had had a bit more time to get used to the features. *(Female, Emergency Care)

It was noted that attention was required when using the new program and that this disrupted their usual clinical thought process. It also led to uncertainty and a lack of confidence in the ability to use DigiTeam.

*[…] but what I don’t trust is if I am able to find the relevant information*,* that I can’t find what is most relevant. It’s not that I don’t trust the system*,* but I don’t trust my ability to retrieve the information. *(Female, Emergency Care)

### Main information elements

The feedback and observations during the development process revealed that the most useful information elements were those that presented the patient’s voice and health problem list and timeline. These information elements were presented on the start page in the final version (Fig. 1) together with the medical history summary that was not tested. This was based on comments and observations from the previous versions of the start page, where the need for scrolling, unnecessary navigation, and too much information led to negative feedback. The initial start page included multiple sections, such as the patient’s voice, About the patient, a GP diagnosis list, a medication list, buttons for lab reports, the note list, and discharge notes, as well as a text-box timeline. The pages that summarized information were also seen as important information elements (see below).

### Person-centered care

#### What matters to the patient and their context

From the beginning, there was a clear vision that the information about what matters to the patient and social and contextual information should have a prominent place. All the versions presented the patient’s voice in a box at the top of the start page, and their social and contextual information was accessed on a separate page via this box or the sidebar menu. All the participants said that they appreciated easy access to the patient’s perspective, as it provided a quick impression of the patient, their perspective on the health situation, and how the health problems affect daily life. The participants explained that this information is uncommon in health records, but when it is present, it is typically in nurses’ notes and rarely in physicians’ notes. It was commented that this information was helpful when prioritizing actions for the patient based on their wishes and what matters to them.

*That part about the patient is really nice when trying to be patient-centered. I don’t know if he wrote it himself or told someone*,* but having it first is great because it reminds us that this is where it starts. What does he want help with*,* and who does he have around him. So*,* that was nice. *(Female, Comprehensive Care Team)

Only one participant did not notice the patient’s voice box, and when the box was pointed out in the interview afterward, he said:

*Here it is*,* I must have overlooked it. But it’s very useful that it is stated so clearly there. I think I got a bit too focused on the note list.* (Male, GP)

Most changes during the development concerned the amount of text about the patient, which was gradually reduced to avoid repetition, as it was asked throughout the tests to shorten the text or use bullet points.

*I have sometimes received letters from patients where they have written what is important to them*,* which is very nice*,* but they can write page after page*,* and it can become overwhelming and difficult to process it. Some short bullet points would probably be best for me. *(Female, Hospital)

#### Supporting integrated and proactive care

A central theme throughout the development process was how to facilitate gaining an overview of all relevant information from several sources while avoiding information overload. The main solution was to present the information chronologically on timelines and in written summaries. This included activities from all involved sources and providing an overview of who was involved at what time, which is needed for integrated care. This method was also crucial for proactive care, as it provided the necessary overview to plan and identify the most beneficial measures.

#### Care information from several sources and professionals

The plan from the start was to include notes from the hospital, GPs, and municipal health and social care services. The participants across the versions appreciated having access to information from several sources, however, they also described challenges such as information overload, missing relevant details, and the need for excessive scrolling in the note list. The participants described that the GP notes provided an overall health status, and the most relevant medical details were found in hospital physicians’ notes such as admission and discharge notes.

Municipal health and social care service notes were first introduced in the fourth version since the patient cases used in the previous versions did not receive these services. One participant described these services as *“a black box”* (Female, Hospital). The participants appreciated having access to notes from municipal health and social care services, as these provided valuable insights into the patients’ daily lives and contributed to a more holistic understanding of their functional status. municipal health and social care serviceIt was also said that it provided a better understanding of potential explanations for adverse health events.

*Yes*,* it can actually be quite useful for those patients who have municipal health and social care services*,* I am not used to that*,* and did not even think at all that I could get [access to notes from municipal health and social care services]. But accessing the report from the night shift and home nursing the day before [a fall can provide details on] if something unusual had happened.* (Female, Emergency Care)

#### Summary of information

From the beginning, the aim was to develop features that presented extracts of relevant information. The final version contained curated summary pages for all areas. The main comments regarding the summary pages were that they supported finding information that otherwise needed to be searched for.

The feedback from the participants was mostly related to the health problem summaries, which underwent the most iterations. The first health problem summaries were introduced in the second prototype version. These were structured with sections including a summary of the status of the problem, listed extracts copied from notes of clinically relevant information, treatment plans, and a list of current and previously involved health professionals or departments. However, these summaries were not used or overlooked by most participants in this version. In the third version, the summaries were made more accessible by relocating the health problem list, from which these summaries are accessed, to a higher position on the start page rather than being on a separate page or lower down.

The participants in the third version commented that the amount of text was overwhelming and that the colors and symbols used were confusing. Suggestions for change were to reduce the details of the disease history or hide it in a collapsable menu and instead provide a clearer summary of the status, changes, decisions, further plans, and other conclusions. The feedback resulted in changing to curated summaries in the fourth version.

*It’s just difficult to decide what is important to read through without knowing where one stands now. I would like to have that first. And based on that*,* I will decide what is important to prioritize when I read through. It is not necessary to read through the entire history that was considered stable 2 years ago and is not followed up further. *(Female, Comprehensive Care Team)

Additionally, the participants liked the overview of the ongoing treatment plan. However, some requested a more precise description and expressed uncertainty about how this section of the summaries was developed.

*This part about further plan*,* that’s the treatment plan*,* that’s what we need*,* either a person or a machine that somehow summarizes it all. This is not found in the records*,* someone has to create it*,* and it’s golden to get it. *(Male, Hospital)

Some highlighted the challenge of obtaining a comprehensive view of the full health history when each health problem was summarized on separate pages. This demanded navigating through all the pages and memorizing essential information from each page. Addressing this feedback, the medical history summary on the start page (1.B2), and the medical history list (Additional file 1, Fig. S4) were added to the final version. These additions were not tested in the formal user tests but were briefly viewed by two previous participants, who commented on the layout and content and preferred the list to start with the latest events first.

The fourth version included a summarized list of municipal health and social care services on the page “About the Patient” and a separate page (Additional file 1, Fig. S5) accessed via the sidebar menu. It was observed that the participants briefly looked at the municipal health and social care service page but preferred to read the summarized information in “About the patient”. One participant talked about why the separate municipal health and social care service page was confusing with different terminology and the organization of the information:

*What does ‘professional note’ mean? It takes some effort to understand what it is*,* it should state a bit more about what I need to know like ‘followed by home nursing for leg ulcer’ […] And then I can click there if I want to know more.* (Female, Hospital)

The summary page presenting the involved health professionals was automatically generated in the second version and provided only a list of names with affiliations. The feedback from one participant highlighted the difficulties in understanding which of them has relevant roles. The third version introduced a manually created list organized as those previously and currently involved. Several participants pointed out the challenge in clinical practice of knowing who is involved and therefore appreciated the summarized page with contact details. However, it was observed that this page was not used by the participants, but it was kept, as it was expected to be relevant in real clinical practice.

Several participants raised concerns about trusting the information provided on the summary pages. Questions about who had summarized the information and when were common questions, mostly related to the health problem summaries. Therefore, the date it was last updated was added to the pages. Hyperlinks to the notes from which the information was extracted were part of the summary page from the beginning, but they were integrated more into the text to show by whom, when, and where the original information was created. Having the medical note displayed on the same page reduced the need for back-and-forth navigation and made it easier to look at the original note. The hyperlinks were highly appreciated by the participants as they provided a way to verify the information from a trusted source.

*I can click* [on the hyperlink] *and double-check* [the information] *in the discharge summary or see where the information comes from*,* and that makes me able to trust it*,* I think. *(Female, Emergency Care)

#### Timelines to visualize the main health problems

The participants across the versions acknowledged the value of visualization on a timeline to comprehend when events occurred. Nevertheless, major changes were made from the initial to the final version.

The main work with the timelines was on presenting the main health problems. The initial timeline with text boxes included parts of medical notes on plans, but it was criticized for lacking context and having a chaotic layout. The timeline concept was supported, and after several iterations, it was revised to visualize healthcare contacts (1.B3), allowing participants to easily see when the main healthcare contacts for each problem had been. It was considered beneficial overall. However, some feedback indicated that some misunderstood the markers, thinking that they displayed all the contacts instead of just the selected ones. Despite this, the decision was made to continue showing only the main contacts, as marking all contacts could result in overcrowding.

*To follow a condition in a way*,* see when it started*,* how many in a way*,* as I interpret it*,* contact there are for that disease. Then you get an impression of how this has developed over time and how often it was in focus. So I found that very useful.* (Male, GP)

Initially, the markers of healthcare contacts had different colors and letters in chronological order to be able to mark them in the notes list. There was no explanation of the colors, and the participants frequently asked about the meaning. The final solution (1.B3) used colors to indicate the source of notes (e.g., GP or discharge note) for the specific contact.

## Discussion

The main finding was that it was possible to develop a tool that was found useful for supporting the workflow in complex patient cases requiring a coordinated longitudinal care process. The main information elements used to gain an overview presented on the start page were the patient’s voice, the medical history summary, and a list of the main health problems with their main healthcare contacts on a timeline. The main information elements for details were presented on summary pages with hyperlinks to original notes for quick navigation to details and verification. The summary pages focused on the patient’s social and family situation, medical history, main health problems, involved health professionals, and medication list.

To the best of our knowledge, no studies have developed a similar solution specifically for complex patient cases. Some solutions have made prototypes developed to help physicians get an overview of information for complex patients like ChartWalk [17], which builds on earlier solutions Doccurate [16] and MedStory [15], and HARVEST [21] which presents information from several sources. Another similar identified solution is the Quebec RSIPA [45] which is a computerized care pathway system specifically designed to meet the needs of frail and disabled older adults in home care and to support person-centered, integrated, and proactive care. The main difference between DigiTeam and these solutions is that the other solutions include a relatively large amount of information on the start page, and they do not prominently present the patient’s voice (what matters to the patient) nor provide summaries in the same manner.

### A quick overview with links to details

The start page in the final version of DigiTeam included only three main information elements. The reason was that the participants, similar to those in previous research [26], emphasized the value of providing the basic information that all physicians need and linking to ways to access details. This study found that having several boxes of information was not user-friendly and did not provide a quick overview, which contradicts other solutions that present several types of features and information on one page [17, 19]. Song et al. [19] presented at least ten elements with little information in each element, and several elements that summarize and visualize information presented by Sultanum et al. [17]. This shows that there are discrepancies in what is best to include on the start page. The variation can be explained by differing views on the purpose of the start page, whether it should offer a broad overview with fewer elements, or present more detailed information across several areas, resulting in a greater number of elements.

Furthermore, it differs in whether further details are displayed on the start page, e.g., as pop-ups or expansions of elements [15–17, 19, 21], or as in DigiTeam, in which clicking on an element moves the user to a new page, where navigation back is needed to see the start page again. To clarify which of these approaches is the optimal solution in different settings, a head-to-head comparison is needed.

Still, a common finding in this and other studies is that the participants want an “immediate context” [15–17, 21] and guidance on where to search next. Thus, it can be recommended that the start page should act as a platform to provide necessary information for shared knowledge about the patient’s situation for all those involved in the care, and provide easy ways to find more detailed information.

### A prominent place for the patient’s voice

The approach of presenting the patient’s voice in a prominent place in the upper left corner on the start page of DigiTeam was based on the principle of allowing ‘what matters’ to the patient to guide decision-making and care delivery [1, 51]. Solutions such as PatientWisdom [36] and the Patient Values Table [35] also present what is important for the patient and report receiving positive feedback, as it facilitates mutual understanding in the care team. Information on what matters to the patient and their preferences and goals is also an important aspect of seeing the whole person to provide contextualized care, which was previously argued by Weiner et al. [52].

It seems that where this information is placed impacts whether it will be noticed and integrated into the workflow. Desai et al. [35] presented the information on a separate page/tab, which was reported to be overlooked. The reason was that viewing such a page was not part of the usual workflow, and doing so required additional time and was difficult to remember. Therefore, it seems reasonable to argue that access to the patient’s voice should not require an extra click [35–37]. Instead, it should be placed so that it is seen immediately to serve as a starting point, which guides instead of disrupts the workflow.

Another aspect is what to include when presenting the patient’s voice. This study, based on previous findings from the same research group [49], made the patient’s voice based on what is important to the patient in both the short and long term and what information health professionals need to know to avoid making the patient repeat themselves. In other solutions, patients were asked to provide information about themselves before a clinical visit [35, 37]. Cusatis et al. [37] reported that this approach allowed patients to reflect on their life priorities and share this with the clinician. Desai et al. [35] reported that such a solution can be useful in difficult situations when patients can no longer express their preferences.

In summary, the findings from previous studies and this study consistently indicate that collecting and displaying an easily accessible brief view of the patient’s perspective should be a central component of any solution to support person-centered care. However, more research is needed to explore how this information can be collected and displayed in health records for all patients where it is essential for care planning. This should consider privacy issues and the varied communication preferences and abilities of these patients.

### Highlighting the main health problems

One of the key principles behind the development of DigiTeam was making clinicians aware of all concurrent health problems and treatment plans. Different solutions with similar goals have been presented previously [16, 19, 21, 24, 45]. These methods range from visualizing the frequency of health problems mentioned in medical notes [16, 21] to list health problems with limited information on the duration of the problem [19, 24, 45]. No solution has been found that presents the main health problems showing different types of medical notes from key healthcare contacts on a timeline. An argument for this solution is that the frequency of documented content presented in Doccurate [16] and HARVEST [21] does not necessarily indicate its relevance [53].

The main advantage of displaying the main health problems as done in the DigiTeam start page was that it helped clinicians obtain a comprehensive view of the patient’s health problems and, at the same time, identify the current active health problems indicated by health care contacts on the timeline.

### Summarizing large amount of information

From the beginning of the development of DigiTeam, extracting and presenting short versions of relevant information was a guiding principle to help clinicians obtain an overview of the patient’s situation. As reported in the results section, several attempts have been made to overcome the challenge of presenting all necessary information using as little text as possible [13]. This study found that curated summaries received more positive responses than extracts did in the form of copies of text from the notes. This can be explained by the clinician’s preference for early and easy access to the most essential information on the current situation and treatment plan [13]. Curated summaries were also presented in the solutions by Sultanum et al. [15–17], who received positive responses since these types of summaries are usually searched for by clinicians. Thus, as expected, based on this study and previous findings, summarizing information is useful for clinicians. In this study, the summaries were manually created, which is a time-consuming task. Automatic solutions like artificial intelligence and large language models can most likely be a sustainable solution in a time-and resource-constrained environment as the healthcare system. However, a scoping review from 2023 [12] found that challenges persist regarding extracting all relevant and accurate information that is based on clinical knowledge and trusting the information. Therefore, more work is needed to make automatically created summaries of high quality and usefulness. The findings in this study can be used to guide the type of information included in such summaries and how to present it.

Nevertheless, the clinicians viewed the summarized information with some uncertainty regarding whether all relevant information was included. Similar comments were reported by Sultanum et al. [15–17] on the automated summaries and text extracts they provided. In these studies, the participants wanted an indication of when the information was last updated, who updated it, and the source. Thus, including references to the information source in the summaries can be recommended to increase trust and provide links to more detailed information.

### Strengths and limitations

The main strength was that several versions were developed and tested iteratively, and a relatively large sample of the intended end-users was included. However, there was relatively limited variation in the physicians’ backgrounds and experiences. Another limitation is that the prototype was tested only in a simulated environment, which cannot capture the complexities of real-world clinical practice. Furthermore, the task used during the testing was to obtain an overview of the patient’s situation without having the opportunity to talk to the patient or relatives, which is something that is usually performed in usual practice. Neither was there detailed information about the clinical context nor what role the participant had beyond using DigiTeam to obtain an overview of the patient’s situation. Thus, the setup did not fully capture the real clinical environment. Although automated log data were not collected, they could have provided insights into actual usage which might have been missed during the observations. The research group’s decisions regarding changes to the prototype were based on their interpretation of the feedback received. Although adhering closely to the initial principles and the feedback, other researchers might have made different choices.

## Conclusion

It was possible to build a tool that supports person-centered, integrated, and proactive care. Physicians found it useful with a simple start page including the main information elements of the patient’s voice and a list of main health problems visualizing key healthcare contacts. This provided an overview that helped understand the patient’s personal and medical situation and supported the workflow by providing different options for going from a focused overview to relevant details. Further research is needed to investigate the effect of the DigiTeam solution.

## Supplementary Information

Below is the link to the electronic supplementary material.

Supplementary Material 1

Supplementary Material 2

## Data Availability

The datasets used and/or analyzed during the current study are available from the corresponding author upon reasonable request.

## References

[1] Berntsen G, Strisland F, Malm-Nicolaisen K, Smaradottir B, Fensli R, Rohne M. The Evidence Base for an Ideal Care Pathway for Frail Multimorbid Elderly: Combined Scoping and Systematic Intervention Review. J Med Internet Res. 2019;21(4):e12517. 31008706 10.2196/12517 PMC6658285

[2] Coulter A, Entwistle VA, Eccles A, Ryan S, Shepperd S, Perera R. Personalised care planning for adults with chronic or long-term health conditions. Cochrane Database Syst Rev. 2015;2015(3):Cd010523. 25733495 10.1002/14651858.CD010523.pub2 PMC6486144

[3] Ho L, Malden S, McGill K, Shimonovich M, Frost H, Aujla N, et al. Complex interventions for improving independent living and quality of life amongst community-dwelling older adults: a systematic review and meta-analysis. Age Ageing. 2023;52(7). 10.1093/ageing/afad132 PMC10378722 37505991

[4] Palmer K, Marengoni A, Forjaz MJ, Jureviciene E, Laatikainen T, Mammarella F, et al. Multimorbidity care model: Recommendations from the consensus meeting of the Joint Action on Chronic Diseases and Promoting Healthy Ageing across the Life Cycle (JA-CHRODIS). Health Policy. 2018;122(1):4–11. 28967492 10.1016/j.healthpol.2017.09.006

[5] Varpio L, Rashotte J, Day K, King J, Kuziemsky C, Parush A. The EHR and building the patient’s story: A qualitative investigation of how EHR use obstructs a vital clinical activity. Int J Med Inf. 2015;84(12):1019–28. 10.1016/j.ijmedinf.2015.09.004 26432683

[6] Zahabi M, Kaber DB, Swangnetr M. Usability and Safety in Electronic Medical Records Interface Design: A Review of Recent Literature and Guideline Formulation. Hum Factors. 2015;57(5):805–34. 25850118 10.1177/0018720815576827

[7] Senathirajah Y, Kaufman DR, Cato KD, Borycki EM, Fawcett JA, Kushniruk AW. Characterizing and Visualizing Display and Task Fragmentation in the Electronic Health Record: Mixed Methods Design. JMIR Hum Factors. 2020;7(4):e18484. 33084580 10.2196/18484 PMC7641790

[8] Eden KB, Totten AM, Kassakian SZ, Gorman PN, McDonagh MS, Devine B, et al. Barriers and facilitators to exchanging health information: a systematic review. Int J Med Inf. 2016;88:44–51. 10.1016/j.ijmedinf.2016.01.004 PMC4778080 26878761

[9] Moy AJ, Hobensack M, Marshall K, Vawdrey DK, Kim EY, Cato KD, et al. Understanding the perceived role of electronic health records and workflow fragmentation on clinician documentation burden in emergency departments. J Am Med Inf Assoc. 2023;30(5):797–808. 10.1093/jamia/ocad038 PMC10114050 36905604

[10] Windle JR, Windle TA, Shamavu KY, Nelson QM, Clarke MA, Fruhling AL, et al. Roadmap to a more useful and usable electronic health record. Cardiovasc Digit Health J. 2021;2(6):301–11. 35265926 10.1016/j.cvdhj.2021.09.007 PMC8890352

[11] Li E, Clarke J, Ashrafian H, Darzi A, Neves AL. The Impact of Electronic Health Record Interoperability on Safety and Quality of Care in High-Income Countries: Systematic Review. J Med Internet Res. 2022;24(9):e38144. 36107486 10.2196/38144 PMC9523524

[12] Keszthelyi D, Gaudet-Blavignac C, Bjelogrlic M, Lovis C. Patient Information Summarization in Clinical Settings: Scoping Review. JMIR Med Inf. 2023;11:e44639. 10.2196/44639 PMC10716777 38015588

[13] Pivovarov R, Elhadad N. Automated methods for the summarization of electronic health records. J Am Med Inf Assoc. 2015;22(5):938–47. 10.1093/jamia/ocv032 PMC4986665 25882031

[14] Bui AA, Aberle DR, Kangarloo H. TimeLine: visualizing integrated patient records. IEEE Trans Inf Technol Biomed. 2007;11(4):462–73. 17674629 10.1109/titb.2006.884365

[15] Sultanum N, Brudno M, Wigdor D, Chevalier F. More text please! Understanding and Supporting the Use of Visualization for Clinical Text Overview. In: Proceedings of the. 2018 CHI Conference on Human Factors in Computing Systems. 2018. pp. 1–13.

[16] Sultanum N, Singh D, Brudno M, Chevalier F. Doccurate: a curation-based approach for clinical text visualization. IEEE Trans Vis Comput Graph. 2018. 10.1109/TVCG.2018.2864905 30136959

[17] Sultanum N, Naeem F, Brudno M, Chevalier F, ChartWalk. Navigating large collections of text notes in electronic health records for clinical chart review. IEEE Trans Vis Comput Graph. 2023;29(1):1244–54. 36166535 10.1109/TVCG.2022.3209444

[18] Alverbratt C, Vikman H, Hjalm Eriksson M, Stattin P, Franck Lissbrant I. Time difference in retrieving clinical information in Patient-overview Prostate Cancer compared to electronic health records. Scand J Urol. 2022;56(2):95–101. 35107408 10.1080/21681805.2021.2014561

[19] Song C, Nakayama M. Implementation of a Patient Summary Web Application According to the International Patient Summary and Validation in Common Use Cases in Japan. J Med Syst. 2023;47(1):100. 37740823 10.1007/s10916-023-01993-6 PMC10517891

[20] Calzoni L, Clermont G, Cooper GF, Visweswaran S, Hochheiser H. Graphical Presentations of Clinical Data in a Learning Electronic Medical Record. Appl Clin Inf. 2020;11(4):680–91. 10.1055/s-0040-1709707 PMC7560537 33058103

[21] Hirsch JS, Tanenbaum JS, Lipsky Gorman S, Liu C, Schmitz E, Hashorva D, et al. HARVEST, a longitudinal patient record summarizer. J Am Med Inf Assoc. 2015;22(2):263–74. 10.1136/amiajnl-2014-002945 PMC4394965 25352564

[22] Liang JJ, Tsou CH, Dandala B, Poddar A, Joopudi V, Mahajan D, et al. Reducing Physicians’ Cognitive Load During Chart Review: A Problem-Oriented Summary of the Patient Electronic Record. AMIA Annu Symp Proc. 2021;2021:763–72. 35308927 PMC8861663

[23] Semanik MG, Kleinschmidt PC, Wright A, Willett DL, Dean SM, Saleh SN, et al. Impact of a problem-oriented view on clinical data retrieval. J Am Med Inf Assoc. 2021;28(5):899–906. 10.1093/jamia/ocaa332 PMC8068438 33566093

[24] Desai AD, Wang G, Wignall J, Kinard D, Singh V, Adams S, et al. User-centered design of a longitudinal care plan for children with medical complexity. J Am Med Inf Assoc. 2020;27(12):1860–70. 10.1093/jamia/ocaa193 PMC7727350 33043368

[25] Pollack AH, Pratt W. Association of Health Record Visualizations With Physicians’ Cognitive Load When Prioritizing Hospitalized Patients. JAMA Netw Open. 2020;3(1):e1919301. 31940040 10.1001/jamanetworkopen.2019.19301 PMC6991320

[26] Jensen LG, Bossen C. Factors affecting physicians’ use of a dedicated overview interface in an electronic health record: The importance of standard information and standard documentation. Int J Med Inf. 2016;87:44–53. 10.1016/j.ijmedinf.2015.12.009 26806711

[27] Tendedez H, Ferrario MA, McNaney R, Gradinar A. Exploring Human-Data Interaction in Clinical Decision-making Using Scenarios: Co-design Study. JMIR Hum Factors. 2022;9(2):e32456. 35522463 10.2196/32456 PMC9123541

[28] Vega-Gorgojo G, Slaughter L, Giese M. Seeing the whole picture: integrated pre-surgery reports with PreOptique. J Biomed Semant. 2019;10(1):5. 10.1186/s13326-019-0197-1 PMC6398264 30832727

[29] Hosseini M, Faiola A, Jones J, Vreeman DJ, Wu H, Dixon BE. Impact of document consolidation on healthcare providers’ perceived workload and information reconciliation tasks: a mixed methods study. J Am Med Inf Assoc. 2019;26(2):134–42. 10.1093/jamia/ocy158 PMC6804409 30566630

[30] Santana MJ, Manalili K, Jolley RJ, Zelinsky S, Quan H, Lu M. How to practice person-centred care: A conceptual framework. Health Expect. 2018;21(2):429–40. 29151269 10.1111/hex.12640 PMC5867327

[31] Schrans D, Avonts D, Christiaens T, Willems S, de Smet K, van Boven K, et al. The search for person-related information in general practice: a qualitative study. Fam Pract. 2016;33(1):95–9. 26787770 10.1093/fampra/cmv099

[32] Senteio C, Veinot T, Adler-Milstein J, Richardson C. Physicians’ perceptions of the impact of the EHR on the collection and retrieval of psychosocial information in outpatient diabetes care. Int J Med Inf. 2018;113:9–16. 10.1016/j.ijmedinf.2018.02.003 29602438

[33] Estiri H, Patel CJ, Murphy SN. Informatics can help providers incorporate context into care. JAMIA Open. 2018;1(1):3–6. 31984312 10.1093/jamiaopen/ooy025 PMC6951901

[34] Cohen DJ, Wyte-Lake T, Dorr DA, Gold R, Holden RJ, Koopman RJ, et al. Unmet information needs of clinical teams delivering care to complex patients and design strategies to address those needs. J Am Med Inf Assoc. 2020;27(5):690–9. 10.1093/jamia/ocaa010 PMC7647291 32134456

[35] Desai AV, Michael CL, Kuperman GJ, Jordan G, Mittelstaedt H, Epstein AS, et al. A Novel Patient Values Tab for the Electronic Health Record: A User-Centered Design Approach. J Med Internet Res. 2021;23(2):e21615. 33595448 10.2196/21615 PMC7929751

[36] Holt JM, Cusatis R, Asan O, Williams J, Nukuna S, Flynn KE, et al. Incorporating patient-generated contextual data into care: Clinician perspectives using the Consolidated Framework for Implementation Science. Healthc (Amst). 2020;8(1):100369. 31445878 10.1016/j.hjdsi.2019.100369

[37] Cusatis R, Holt JM, Williams J, Nukuna S, Asan O, Flynn KE, et al. The impact of patient-generated contextual data on communication in clinical practice: A qualitative assessment of patient and clinician perspectives. Patient Educ Couns. 2020;103(4):734–40. 31744702 10.1016/j.pec.2019.10.020

[38] Van Citters AD, Gifford AH, Brady C, Dunitz JM, Elmhirst M, Flath J, et al. Formative evaluation of a dashboard to support coproduction of healthcare services in cystic fibrosis. J Cyst Fibros. 2020;19(5):768–76. 32354650 10.1016/j.jcf.2020.03.009

[39] Taxter A, Johnson L, Tabussi D, Kimura Y, Donaldson B, Lawson E, et al. Co-design of an electronic dashboard to support the coproduction of care in pediatric rheumatic disease: human-centered design and usability testing. J Particip Med. 2022;14(1):e34735. 35133283 10.2196/34735 PMC9077505

[40] Van Citters AD, Holthoff MM, Kennedy AM, Melmed GY, Oberai R, Siegel CA, et al. Point-of-care dashboards promote coproduction of healthcare services for patients with inflammatory bowel disease. Int J Qual Health Care. 2021;33(Supplement2):ii40–7. 34849970 10.1093/intqhc/mzab067

[41] Seljelid B, Varsi C, Solberg Nes L, Stenehjem AE, Bollerslev J, Borosund E. Content and system development of a digital patient-provider communication tool to support shared decision making in chronic health care: InvolveMe. BMC Med Inf Decis Mak. 2020;20(1):46. 10.1186/s12911-020-1065-8 PMC7057594 32131808

[42] Schmajuk G, Nasrallah C, Berrean B, Prugh J, Wilson C, Hamblin A, et al. A step-by-step roadmap for the development and deployment of an electronic health record sidecar application that tracks patient outcomes: The RA PRO dashboard. Digit Health. 2024;10:20552076241288739. 39421306 10.1177/20552076241288739 PMC11483702

[43] Brown EG, Schleimer E, Bledsoe IO, Rowles W, Miller NA, Sanders SJ, et al. Enhancing Clinical Information Display to Improve Patient Encounters: Human-Centered Design and Evaluation of the Parkinson Disease-BRIDGE Platform. JMIR Hum Factors. 2022;9(2):e33967. 35522472 10.2196/33967 PMC9123539

[44] Mohindra NA, Garcia SF, Kircher S, Barnard C, Perry LM, Lyleroehr M, et al. Development of an electronic health record-integrated patient-reported outcome-based shared decision-making dashboard in oncology. JAMIA Open. 2024;7(3):ooae056. 39049991 10.1093/jamiaopen/ooae056 PMC11268523

[45] Dubuc N, Brière S, Corbin C, N’Bouke A, Bonin L, Delli-Colli N. Computerized Care-Pathways (CCPs) System to Support Person-Centered, Integrated, and Proactive Care in Home-Care Settings. Inf Health Soc Care. 2021;46(1):100–11. 10.1080/17538157.2020.1865969 33406972

[46] Gulliksen J, Göransson B, Boivie I, Blomkvist S, Persson J, Cajander Å. Key principles for user-centred systems design. Behav Inform Technol. 2003;22(6):397–409.

[47] Rijken MSV, van der Heide I, Hujala A, Barbabella F, van Ginneken E, et al. How to improve care for people with multimorbidity in Europe? World Health Organization, Regional Office for Europe; 2017. 29144712

[48] Shneiderman B, editor. The eyes have it: a task by data type taxonomy for information visualizations. Proceedings 1996 IEEE Symposium on Visual Languages; 1996 3–6 Sept. 1996.

[49] Silsand L, Severinsen GH, Berntsen GR, Steinsbekk A. How to Represent the Patient Voice in the Electronic Health Record? Stud Health Technol Inf. 2023;302:187–91. 10.3233/SHTI230100 37203644

[50] Ingemann IK. Brukbarhetstesting av et digitalt verktøy for samhandling om personer med store og sammensatte behov [Master’s thesis]: Norwegian University of Science and Technology; 2022.

[51] Berntsen GK, Gammon D, Steinsbekk A, Salamonsen A, Foss N, Ruland C, et al. How do we deal with multiple goals for care within an individual patient trajectory? A document content analysis of health service research papers on goals for care. BMJ Open. 2015;5(12):e009403. 26656243 10.1136/bmjopen-2015-009403 PMC4679896

[52] Weiner SJ. Contextualizing care: An essential and measurable clinical competency. Patient Educ Couns. 2022;105(3):594–8. 34158194 10.1016/j.pec.2021.06.016

[53] Colicchio TK, Dissanayake PI, Cimino JJ. Physicians’ perceptions about narrative note sections format and content: A multi-specialty survey. Int J Med Inf. 2021;151:104475. 10.1016/j.ijmedinf.2021.104475 33975266

