# Supplementary material for: Development of a digital tool to support overview in complex patient cases: Which information elements support the clinical workflow?
Source: BMC Health Serv Res. 2026 Apr 11;26:734. doi: 10.1186/s12913-026-14466-6 (PMC13195975; doi:10.1186/s12913-026-14466-6)

# Additional file 1: Figures of the prototype

*Figure S3: The note list is on the left side and the medical note is displayed on the right side. On top of this page is a filter- and search function, and below these is a density graph showing when the note has been written.*

*
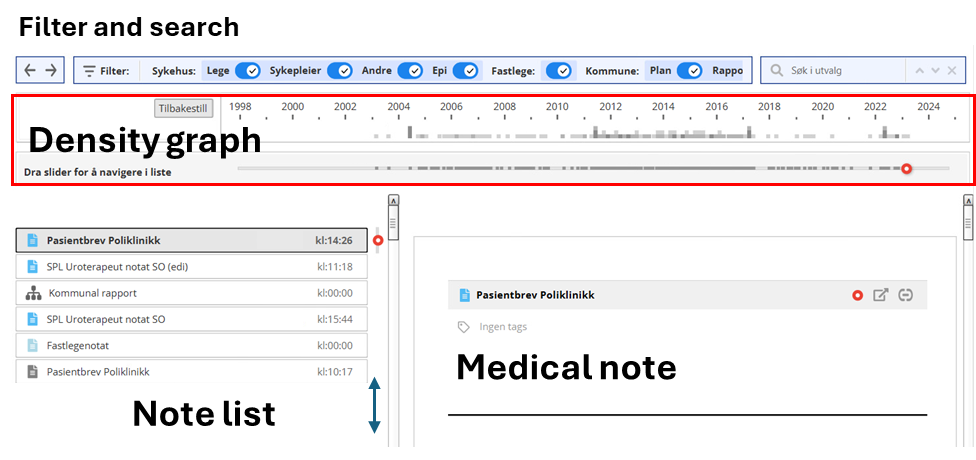
*

*Figure S4: The page with the full medical history can be accessed via the sidebar menu or through the box with the summarized disease history. This page includes health problems, diagnoses, and health events listed chronologically. The links provide access to the medical note or the health problem summary page.*

*
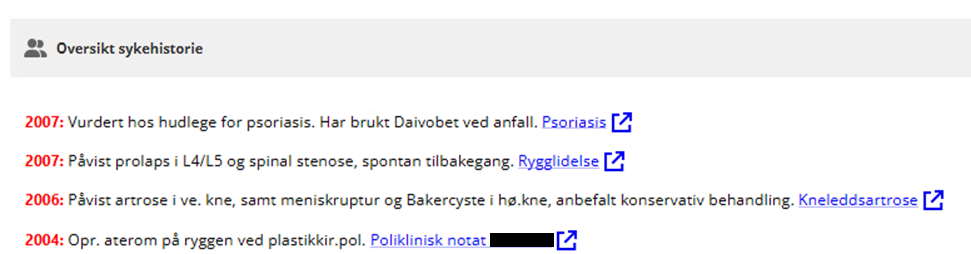
*

*Figure S5: The list of current, past, and PRN medicine report dates for start, change, or discontinuation with hyperlinks to the medical note to access more information.*


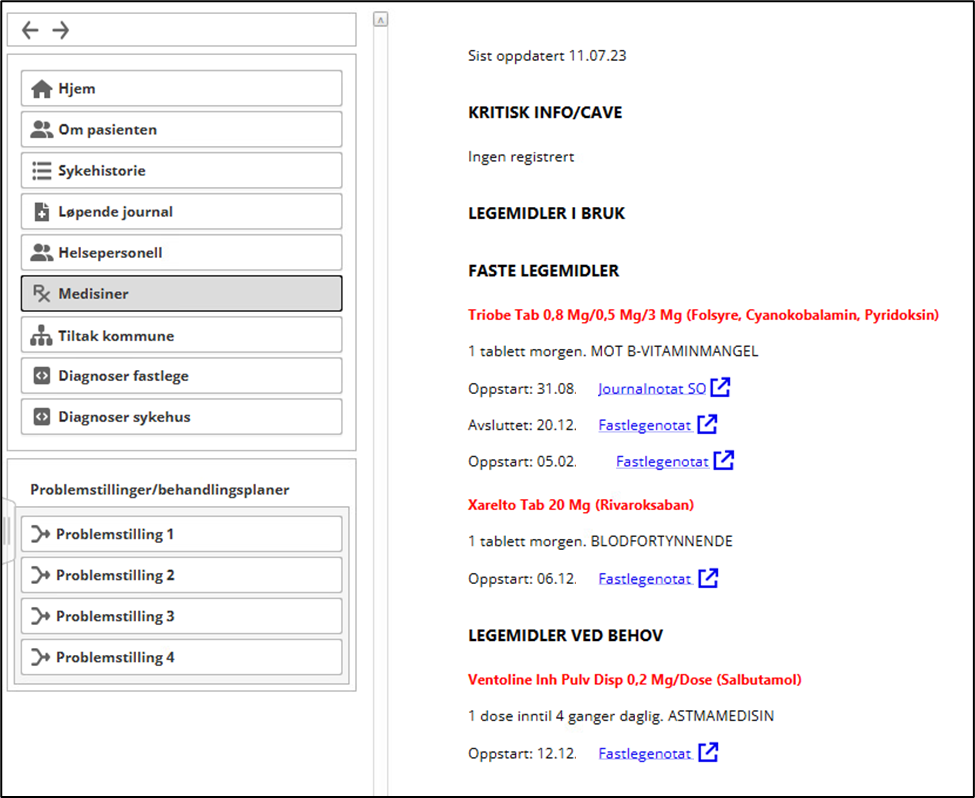


*Figure S6: The municipal health service page shows the care area plan with timelines, visualizing the plan's start, change, and discontinuation.*


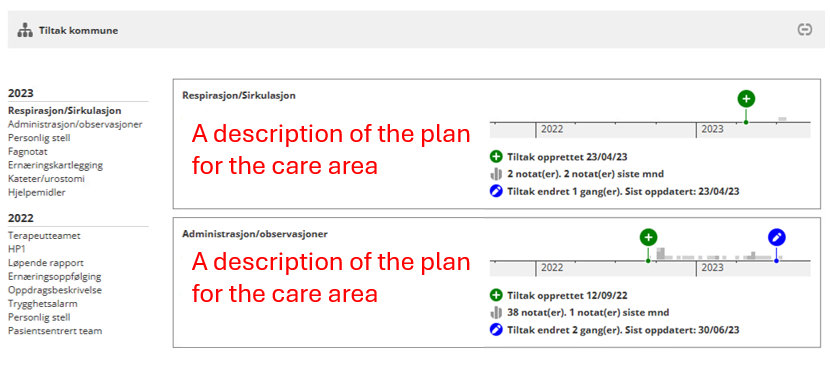

Supplement: Supplementary file 1 — Supplementary Material 1 [file 12913_2026_14466_MOESM1_ESM.docx]
