# Supplementary material for: Development of a digital tool to support overview in complex patient cases: Which information elements support the clinical workflow?
Source: BMC Health Serv Res. 2026 Apr 11;26:734. doi: 10.1186/s12913-026-14466-6 (PMC13195975; doi:10.1186/s12913-026-14466-6)
Supplement: Supplementary file 2 — Supplementary Material 2 [file 12913_2026_14466_MOESM2_ESM.docx]

Additional file 2: The interview guide

**Overall** **impression**

- What is your overall impression of the program?

**Solving the task**

- How did you perceive DigiTeam’s ability to support understanding the patient’s situation and current challenges?
- What do you think about DigiTeam’s ability to provide information to understand what needs to be followed up and further actions?
- Which features did you use to solve the task?

Ask about the use and experience of features for:

- Municipal health services
- Medication list with hyperlinks
- Summary pages
- Timeline
- Patient’s perspective

**Usability and Experience of the Program**

- How intuitive did you find and navigate DigiTeam in terms of finding the information you needed to solve the task?
- Was there anything difficult to understand or find in DigiTeam? What could have been done differently?
- What do you think about the trustworthiness of the information you accessed in DigiTeam?
- Now that you have tested the tool and received some training, if you were to do this again with the experience you have now, how would you approach solving the task?
- What do you think about the introduction to DigiTeam in the beginning? Do you think you could have learned the program on your own, or was the introduction necessary?
